# Centrioles without microtubules: a new morphological type of centriole

**DOI:** 10.1242/bio.036012

**Published:** 2018-07-11

**Authors:** Rustem Uzbekov, Anastasiia Garanina, Christophe Bressac

**Affiliations:** 1 Department of Microscopy, University of Tours, Tours 37032, France; 2 Faculty of Bioengineering and Bioinformatics, Moscow State University, Moscow 119992, Russia; 3 Institute of Research on Insect Biology, IMIP research team UMR CNRS 7261, University of AQ1 Tours, Tours 37200, France

**Keywords:** Centriole, Centrosome, Cilia, Insect, Parasitoid wasp, Microtubules

## Abstract

The centrosome is the organizing center of microtubules in the cell, the basis for the origin of cilia and flagella and a site for the concentration of a regulatory proteins multitude. The centrosome comprises two centrioles surrounded by pericentriolar material. Centrioles in the cells of different organisms can contain nine triplets, doublets or singlets of microtubules. Here, we show that in somatic cells of male wasp larvae *Anisopteromalus calandrae*, centrioles do not contain microtubules and are composed of nine electron-dense prongs, which together form a cogwheel structure. These microtubule-free centrioles can be the platform for procentriole formation and form microtubule-free cilia-like structures. In nymph and imago cells centrioles have a microtubule triplet structure. Our study describes how centriole structure differs in a development-stage-dependent and a cell-type-dependent manner. The discovery of a centriole without microtubules casts a new light on the centriole formation process and the evolution of this organelle.

## Abstract (precis)

**Summary:** Centrioles without microtubules in larvae cells of parasitoid wasps duplicate and form primary cilia without microtubules, in nymph and imago cells centrioles acquire a ‘normal’ structure with nine microtubules triplets.

## INTRODUCTION

The ultrastructure of centrioles was described for the first time in the middle of the 1950s, when the arsenal of cell biology methods was enhanced by electron microscopy (Fawcett and Porter, 1954; Burgos and Fawcett, 1955; Bernhard and de Harven, 1956). The first descriptions could not correctly establish the three-dimensional structure of this organelle, but after the improvement of sample preparation methods and staining, it was shown that the centriole consisted of nine microtubules (MT) triplets (Brinkley and Stubblefield, 1970; Wheatley, 1982). This paradigm has remained for a long time, but the gradual accumulation of new data has shown that, at least in some types of insect cells, the centriole has a different structure and consists of MT doublets (Riparbelli et al., 2010). Also, in one-cell embryos of the model nematode *Caenorhabditis elegans*, the centriole may consist of nine singlets of MT (O'Toole et al., 2003; Pelletier et al., 2006), differing from the basal body in sensitive neurons where there are nine doublets of MT (Sulston et al., 1980). Soon after formation the basal bodies containing the doublets of MT are disassembled at the base of the cilia (Serwas et al., 2017; Nechipurenko et al., 2017). Thus, the possible diversity of the structure of centrioles in animals was postulated (Azimzadeh and Bornens, 2004; Gupta and Kitagawa, 2018).

Another observation concerning the structural diversity of centrioles was that somatic cell centrioles in *Drosophila* consist of doublets and germ cell line centrioles consist of MT triplets (Gottardo et al., 2015). In the cell cycle of vertebrates there is also a stage when young procentrioles consist of MT doublets, but this stage is very short and occurs near the beginning of procentriole formation (Guichard et al., 2010).

In present study it has been shown that the structure of centrioles at different stages of development of the organism can differ even more dramatically than previously shown for either *Drosophila melanogaster* and *C*. *elegans*; centrioles in the insect *Anisopteromalus calandrae* larvae did not contain MT. As yet, centrioles without MT have not been described. It appears surprising to propose such a structure because it has been assumed that MT are an integral part of centrioles. The present study casts a new light on the centriole formation process and confirms the prediction of Riparbelli and co-authors that the insect centriole is a ‘land of discovery’ (Riparbelli et al., 2010).

## RESULTS AND DISCUSSION

### Ultrastructure of male centrioles in three species of wasps

**Fig. 1.**
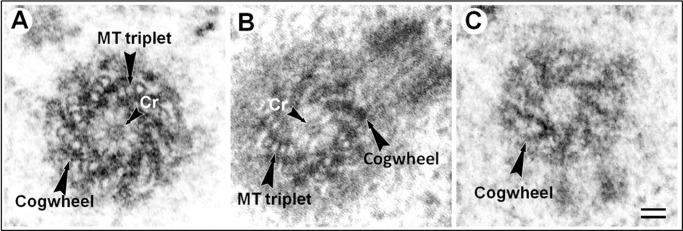
**Centriole structure in larval cells of three species of wasps.** (A) *N. vitripennis*, (B) *C. congregata*, (C) *A. calandrae*. Prongs of the cogwheel are visible between triplets in *Nasonia* and *Cotesia* centrioles, the *Anisopteromalus* centriole has a cogwheel without MT. View from the distal end of the centriole. Cr, cartwheel structure. Scale bar: 50 nm.

**Fig. 2.**
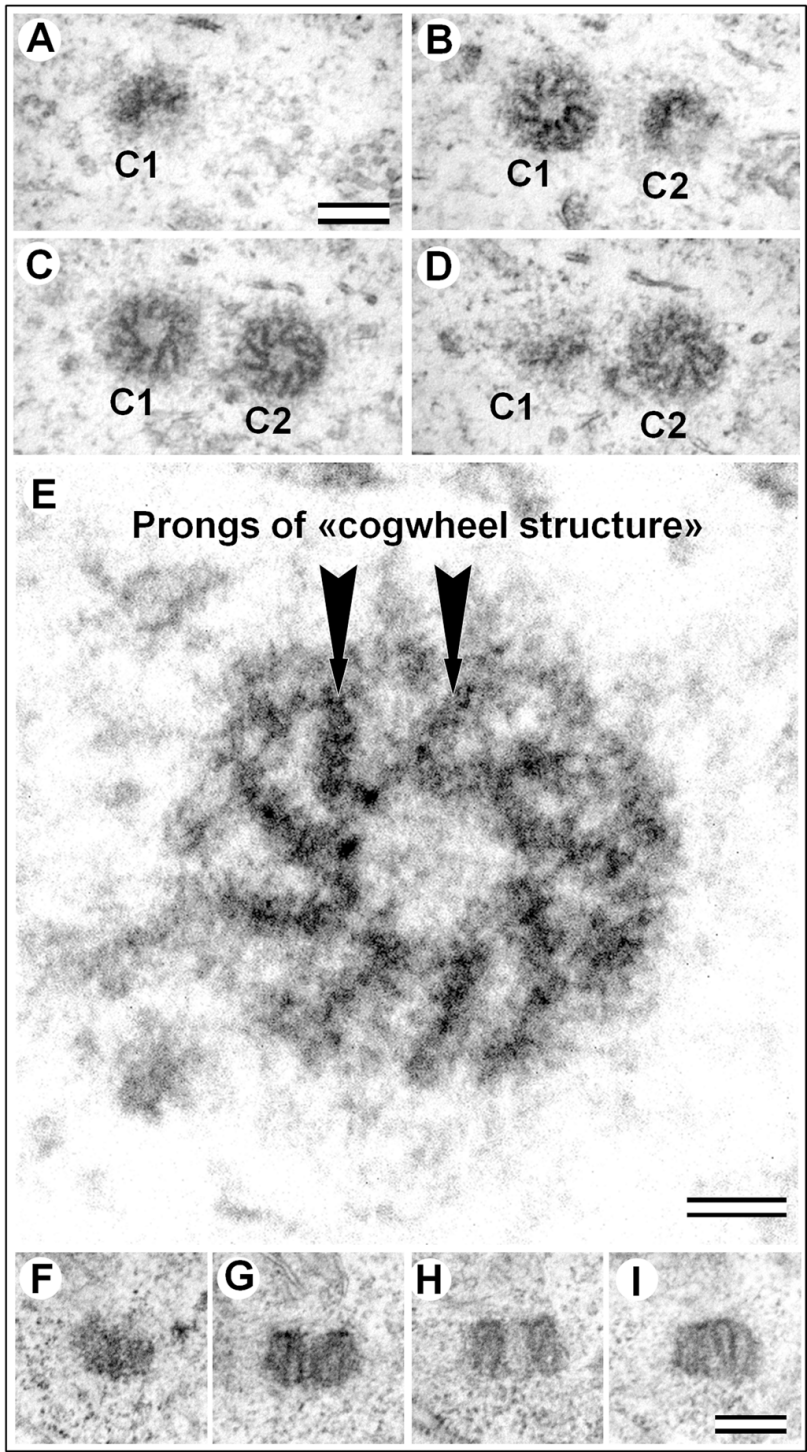
**Fine centriole structure in the epithelial somatic cell of male *A. calandrae* larvae.** (A–D) Four consecutive serial cross-sections of two centrioles, view from the distal ends of the both centrioles. (E) Cross-section of the centriole from panel C (C2) at high magnification. (F–I) Four consecutive serial sections parallel to the centriole axis. C1, centriole 1; C2, centriole 2. Scale bars: 200 nm for A–D,F–I. 50 nm for E.

We investigated cells of three species of wasps: *Cotesia congregata*, *Nasonia vitripennis* and *A. calandrae*. Only males were considered due to the fact that they are haploid in Hymenoptera. Centrioles and cilia of adult and nymphs cells had compositions typical for other insects. Centrioles contained triplets and cilia doublets of MT (Fig. S1). In larvae cells, centrioles of *C. congregata* and *N. vitripennis* had MT triplets, too (Fig. 1A,B). Surprisingly, centrioles of *A. calandrae* larvae somatic cells (trophocytes and hypodermal cells were studied) had no MT triplets (Figs 1C and 2; Fig. S2). The structure of centrioles in trophocytes and hypodermal cells was identical. The wall of the centrioles consisted of nine prongs of electron-dense material, which were distributed by ninefold central symmetry and occupied the entire length of the centrioles. We propose to call this the cogwheel structure, and we call the nine components that formed this structure the prongs of the cogwheel. Centrioles with very similar morphology, and without MT, were found after centrosome isolation from young *Drosophila* larvae (Gopalakrishnan et al., 2010, Fig. 1); however, the authors did not pay much attention to this observation. It must be noted, that between triplets of MT in the centrioles of *N. vitripennis* and *C. congregata* larvae cells (Fig. 1) as well as in *Drosophila* between MT doublets in somatic cells and between MT triplets in primary spermatocytes (Gupta and Kitagawa, 2018), the prongs were arranged similarly to the prongs of the cogwheel structure in *A. calandrae*.

The direction of the prongs' rotation in *N. vitripennis* and *C. congregata* was the same as for MT triplets (clockwise when viewed from the distal end of centriole).

Because of the specificity of *N. vitripennis* and *C. congregata* larvae sample preparation, they were older than *A. calandrae* larvae when observation was first possible. Indeed, *A. calandrae* larvae develop outside from their host (ectoparasitoid) so they can be observed continuously from the egg to pupation; both other species are endoparasitoid, developing inside the body or the puparium of the host. Therefore, we decided to more precisely investigate the structure of centrioles from *A. calandrae* during the course of individual development in pupae and adult insects, in order to understand at what stage of development the centrioles change their structure.

**Fig. 3.**
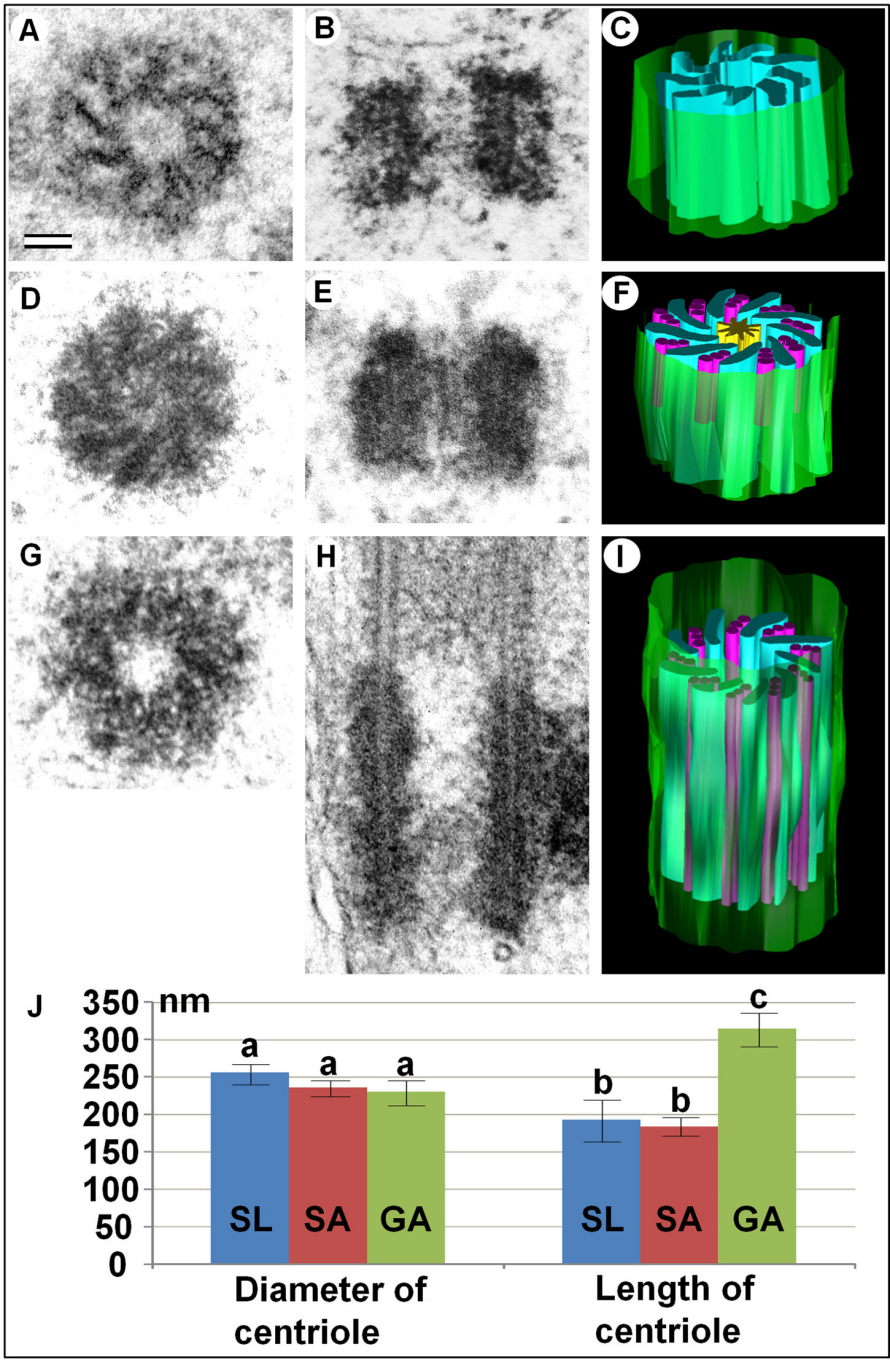
**Comparative analysis of centriole structure in *A. calandrae* larval cells (A–C) and epithelial somatic (D–F) and generative (G–I) cells (spermatids) of imago.** Cross sections (A,D,G), longitudinal sections (B,E,H) and 3D reconstructions (C,F,I) of three morphological types of centrioles are shown. (J) Histogram of centriole diameter and length distribution for somatic larval (SL), somatic adult (SA) and generative adult (GA) cells of *A. calandrae.* The diameter of centrioles in somatic and male germ cells doesn't differ significantly (letter a on the left side; *t*-test, *P*>0.01), while the length of centrioles in somatic cells is much less than in male germ cells (letters b and c on the right part; *t*-test, *P*<0.01). Scale bar: 50 nm.

In contrast to larval cells, centrioles from *A. calandrae* nymph and adult cells had triplets of MT in the wall of the centriolar cylinder and the wall of the primary cilium contained doublets of MT (Fig. S1; Fig. 3). Centriole length in somatic cells (trophocytes and hypodermal cells) was 190±15 nm (*N*=9, min=169 nm, max=213 nm) and the diameter was 244±9 nm (*N*=9, min=231 nm, max=259 nm). In male germ cells (spermatids), centriole length was 317±26 nm (*N*=14, min=264 nm, max=360 nm) and the diameter was 231±14 nm (*N*=18, min=201 nm, max=253 nm). Consequently, the diameter of the centrioles did not differ significantly in somatic and male germ cells (*t*-test, *P*=0.025), and the length of the centrioles in the male germ cells was significantly larger (*t*-test, *P*=1.06E-11).

The central hub of the cartwheel structure was always clearly visible in somatic cells of *A. calandrae* pupae and in imago, but was absent in male germ cells (Fig. 3).

### Ultrastructure of *A. calandrae* male larvae centrioles

A haploid epithelial cell in the G_1_-phase of the cell cycle harbors two centrioles, which usually are oriented parallel to each other. Analysis of ultrathin cross-sections of these centrioles showed that they did not contain MT (Figs 2,3).

The diameter of the centrioles was on average 255±14 nm (*N*=32, min=221 nm, max=277 nm). In longitudinal sections of the centrioles, the centriole diameter was seen to be nearly identical at both ends (Fig. 2F–I).

The length of the centrioles was more variable: 192±24 nm (*N*=26, min=167 nm, max=252 nm). The structure of both centrioles in the same cell did not have any obvious differences, it follows that the mother and daughter centrioles were morphologically indistinguishable from each other.

The difference in centriole length for the two centrioles in the same cell was 5±3 nm (for 14 cells where both centrioles could be measured; *t*-test, *P*=0.77), which is at the limit of measuring accuracy.

The difference in centriole diameter was also at the limit of measuring accuracy and was 6±4 nm (for six cells where both centrioles could be measured; *t*-test, *P*=0.63).

So, we can conclude that two centrioles of the same cell didn't differ in length and diameter. The centriole wall was comprised of nine prongs of cogwheel that were submerged in a less electron-dense matrix (Fig. 2E).

Prongs of the *A. calandrae* cogwheel structure have a length (in cross-section) of around 100 nm and a thickness of 30 nm, with an inclination angle of approximately 30°. The thickness of the cogwheel wall was approximately 80 nm. The prongs were connected by their bases to form a wall of the inner lumen with a diameter of around 75 nm. The ends of the prongs were bent toward the centriole center. The internal structure of the prongs was not uniform, it consisted of tightly-packed globules with a diameter of 8–10 nm (Fig. 2E). In longitudinal sections of the centriolar cylinder, it was seen that the centrioles contained prongs throughout their entire length. This observation was confirmed by the images of cross-sections; the prongs were visible in all sections of the centrioles. Thus, the overall dimensions of prongs were approximately 190×100×30 nm.

The distance between the two centrioles (*N*=12) varied over a wide range (min=75 nm, max=1246 nm). In six cells, the distance was between 100 nm and 250 nm, but in two cells it was less than 100 nm (Fig. 2) and in four cells it was greater than 300 nm. In a cell with the maximum inter-centriole distance, a mitochondrion was found between the two centrioles.

Both mature centrioles in the cells of *A. calandrae* larvae were identical in size and structure, contrary to vertebrate centrioles where old mother centrioles can have some additional structures, such as distal appendages and sub-distal appendages (Wheatley, 1982) otherwise known as pericentriolar satellites (Vorobjev and Chentsov, 1982).

### Duplication of MT-free centrioles

**Fig. 4.**
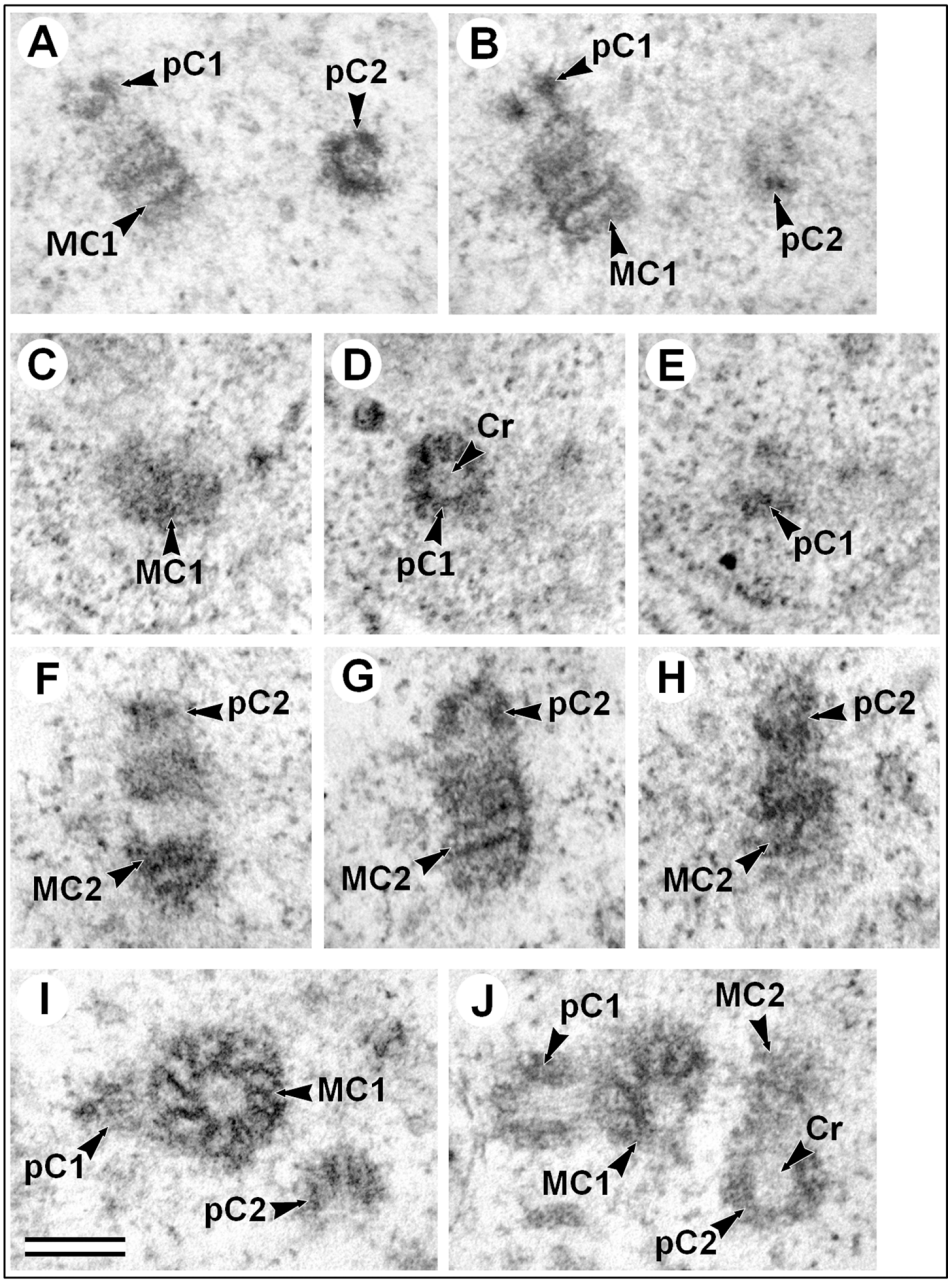
**Procentriole structure in the somatic cells of male *A. calandrae* larvae.** (A,B) Two consecutive ultrathin sections of early procentrioles, longitudinal (pC1) and cross-sections (pC2). (C–E) Three consecutive ultrathin serial sections, the perpendicular (pC1) to the central axis of the procentriole plane. (F–H) Three consecutive ultrathin serial sections, the longitudinal to the central axis of the procentriole plane, in the middle ‘age’ of procentrioles and in the same cell as shown in C–E. (I,J) Two consecutive ultrathin sections of late procentrioles, longitudinal (pC1) and oblique (pC2) to the central axis of the procentriole plane. Cr, cartwheel structure; MC, mother centriole; pC, procentriole. Scale bar: 200 nm.

Duplication of MT-free centrioles occurs in the usual manner (Fig. 4). Procentrioles are formed near the lateral surface of both mother centrioles, perpendicular to their lateral surface. Since the diameter of procentrioles was similar to the length of the mother centrioles, the procentrioles were equidistant from both ends of the mother centriole (Fig. 4B,G).

Changes in the morphology of procentrioles in the successive stages of formation were studied using serial longitudinal- and cross-section sections. Maximum information was obtained from the analysis of cells, in which one of the procentrioles was cut perpendicular to the axis and the second procentriole was cut parallel to the axis (Fig. 4).

Since procentrioles grow during the cell cycle, procentrioles of different lengths were found in various cells, ranging from 62 nm to 167 nm (*N*=17). The diameter of procentrioles was also variable, ranging from 145 nm to 195 nm (*N*=21). However, within a single cell both the diameter and the length of two procentrioles associated with different parent centrioles did not differ.

An internal lumen with low electron density was clearly visible along the entire length of the centrioles. This lumen had an average diameter in mother centrioles of 74±6 nm (*N*=34) and in procentrioles of 66±5 nm (*N*=22). This difference was statistically significant (*P*=1.1E-05) and especially noticeable in young procentrioles (Fig. 4A,B).

### Centriole polarity and cilia formation

Centrioles are polar structures with morphologically different ends: the proximal end of the centriole in model objects is directed towards the nucleus; the distal end of the centriole in model objects is directed away from the nucleus, toward the cell membrane (Wheatley, 1982; Uzbekov and Prigent, 2007). Usually two centrioles face each other with their proximal ends, as originally the proximal end of the procentriole is connected to the lateral surface of the mother centriole and separates from it only after mitosis. Another feature characterizing the polarity of centrioles is a twist of the MT triplets; when the centriole is viewed from the distal end, the vector extending from ‘MT A’ to ‘MT C’ in the triplets is always twisted clockwise (Uzbekov and Prigent, 2007).

In vertebrates, the length of the centriole is usually significantly greater than the diameter. Procentrioles are formed near the proximal end of the mother centriole and grow by elongating at their distal end. Thus in vertebrates the proximal and the distal ends in duplicated centrioles are easy to determine.

However, in cells of *A. calandrae* larvae, the average diameter of the procentrioles (169±19 nm, *N*=21) was almost equal to the average length of the mother centriole (192±28 nm, *N*=26). In the cases where the diameter of the procentriole was smaller than the length of the mother centriole, the procentriole was positioned centrally. Due to that, (using only ultrastructural data) it was not possible to determine proximal-distal polarity of the mother centriole.

Nevertheless, the prongs of the cogwheel have a characteristic twist, which introduces an inherent asymmetry to the entire centriole. Thus, although the direction of the twist still needs to be elucidated, it could likely follow the pattern of the MT triplets in centrioles that contain MT (Fig. 1), where the twist is clockwise when viewed from the distal end of the centriole (Uzbekov and Prigent, 2007).

### Centrioles without MT can build cilia-like structures

In addition to the specific location of the procentriole on the surface of the mother centriole relative to its proximal end, there is an important functional difference between the two ends of the centriole. The cilium or flagellum is always formed at the distal end of the centriole (Wheatley, 1982). We found that in cells of *A. calandrae* larvae, centrioles can form structures that are analogous to primary cilia. However, these ‘primary cilia-like structures’, similar to the centrioles, do not contain MT.

**Fig. 5.**
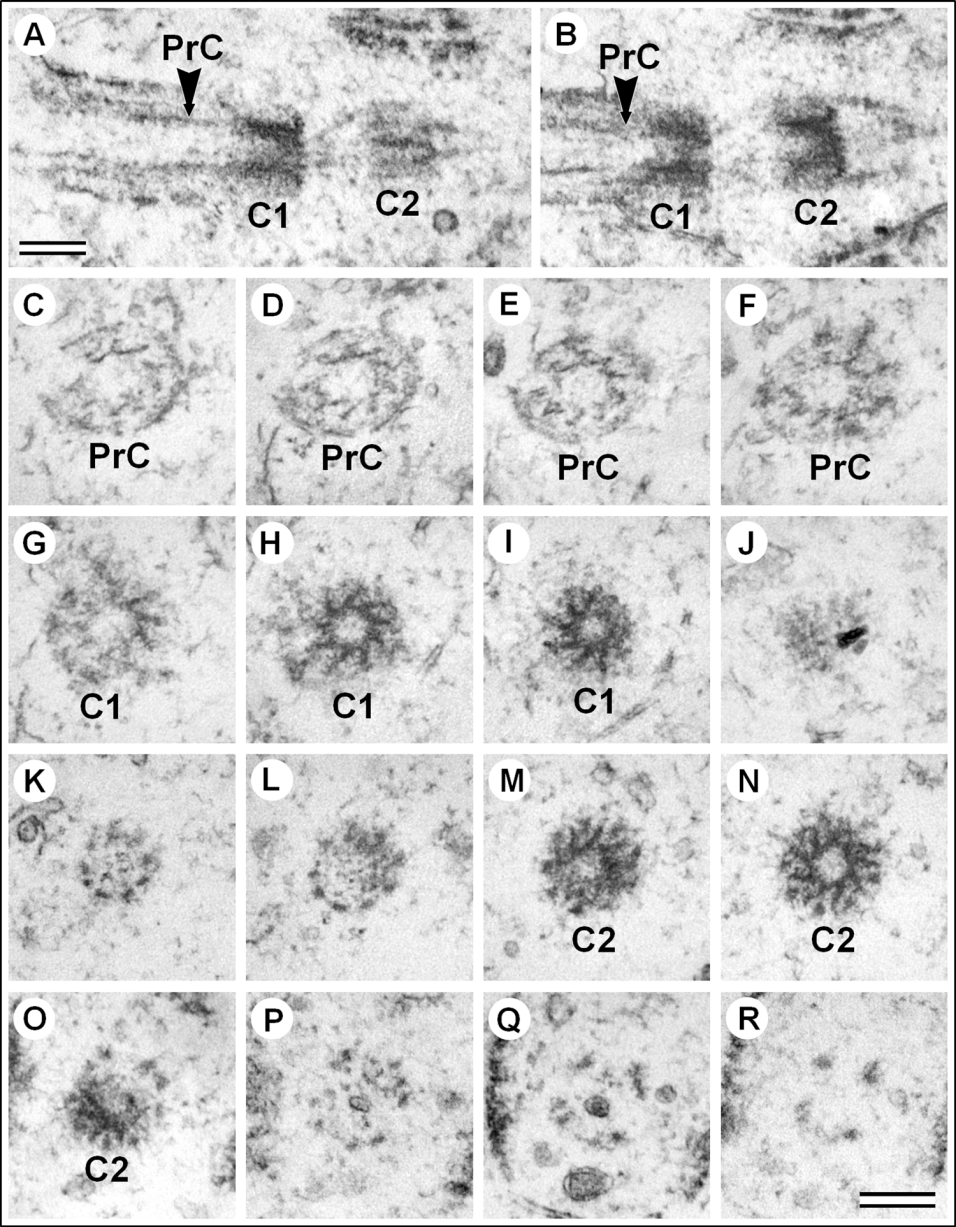
**Serial sections of centrioles and ‘primary cilia’ in the cells of male *A. calandrae* larvae.** (A,B) Two longitudinal consecutive sections of centrioles and cilia; (C–R) 16 consecutive perpendicular sections of cilia and centrioles, the view from the top of the cilia (view from the distal end of the centriole). The identical direction of the twist in the cogwheel prongs shows that the axis of each of the two centrioles is oriented in the same direction. Primary cilia, like the centrioles, have 9-order symmetry and do not have MT. C1, centriole 1; C2, centriole 2; PrC, primary cilia. Scale bars: 200 nm.

In elucidating the orientation of the cross-sections to the axis of the cilium-centriole complex, we could establish the direction in which the nine prongs twisted. Fig. 5 shows longitudinal and serial cross-sections through the cilium-centriole complex. Since the initial cross-sections were from the top of the cilia, we observed the first centriole from the distal end (Fig. 5H,I). From this view, the prongs of the cogwheel were twisted clockwise, which is the same direction as twisted MT triplets of classical centrioles. The second centriole exhibited the same direction of twist in the prongs (Fig. 5M,N), so it was oriented in the same direction. Thus, in the complex the proximal end of the first centriole was connected to the distal end of the second centriole. As in a ‘classical’ primary cilium that contains MT, correct radial symmetry of ‘axoneme’ was preserved only near the centrioles and then it was disturbed (Fig. 5C–F). Structures that connect to the proximal end of the mother centriole and the distal end of the daughter centriole (Fig. 5A,K,L) appear to be rootlets, but we did not find a transverse striation, which is typical of these structures usually associated with centrioles.

### Abilities of MT-free centrioles

One of the principal functions of the centrosome is to organize the MT system in the cell (Wheatley, 1982; Uzbekov and Alieva, 2008). Centrosomes can produce four different structures containing MT; procentrioles, the radial system of MT in interphase cells, the mitotic spindle and cilia or flagella (Uzbekov and Alieva, 2008; Uzbekov, and Alieva, 2013). In the present study we found that centrioles without MT retained almost all of the potential activities of normal centrioles. They could form centrosomes with two centrioles and produce procentrioles. So in larvae morphologically immature centrioles are able to duplicate and transfer the basic structure of this organelle from one generation of cells to another. This process can be compared with the axolotl (*Ambystoma tigrinum* larvae) life cycle, in which reproduction can occur at the larval stage (Weismann, and von Chauvin, 1865).

Centrosomes consisting of centrioles not containing MT are not active centers of MT nucleation in larvae cells. However, we also did not observe the radial system of MT associated with the centrosome in the cells of nymphs and adults. Earlier, function of interphase radial MT network formation was demonstrated to be not clearly expressed in many cell types with normal centrioles, for example, in osteocytes of vertebrates (Uzbekov and Benhamou, 2014).

Previously, it was shown that the cell culture of *Drosophila* without centrioles was able to continue cell division (Szöllösi et al., 1972, 1986). Moreover, the acentriolar DSas-4 mutant flies developed up to the adult stage (Basto et al., 2006). Later, a more detailed study of such mutants showed that this development cannot be considered normal, since it was found out that acentrosomal *Drosophila* epithelial cells exhibit abnormal cell division leading to cell death and compensatory proliferation (Poulton et al., 2014). Thus, although mitotic cell cycles can take place in the absence of centrioles, this is an error-prone process that opens the fly up to developmental defects (Lattao et al., 2017). Centrinone, a reversible inhibitor of Polo-like kinase 4 (Plk4), caused centrioles and centrosome loss in non-cancerous cell lines and irreversibly arrested cells in the G_1_-phase of the cell cycle in a p53-dependent manner. In contrast, cancer-derived cell lines could proliferate indefinitely after centrosome loss, but mitotic duration was increased (Wong et al., 2015).

However, one of the activities of the centrosome is absolutely impossible without centrioles – the formation of cilia (for movement or sensitive function) and flagella – because only centrioles can function as basal bodies (Wheatley, 1982).

Here, we describe the formation of atypical cilia that do not contain MT. However, it is not currently clear whether these cilia have any function.

During the transition from larva to nymph and subsequently to adult, the morphology of centrioles in *A. calandrae* changes. The most important modification is the appearance of MT. The reason for this morphological change of their centrioles could be that the later developmental stages utilize motile cilia by somatic cells and flagella by spermatozoa, which both require MT for their motion.

It is possible that during individual cell development, cells selectively activate a specific set of genes that regulate centriole morphology and the appearance of MT triplets. The difference in the length of centrioles in somatic and male germ cells (Fig. 3) may reflect the difference in motor functions of spermatozoa flagella and cilia of somatic cells.

For a long time it was thought that the structure of centrioles in different cells of one organism could vary only by the presence or quantity of different centriole-associated structures, such as sub-distal appendages (pericentriolar satellites), the primary cilium or striated rootlets. However, it was shown that MT doublets were present in *Drosophila* centriolar cylinders in somatic cells, and MT triplets were present in generative cells (Tates, 1971; Gottardo et al., 2015).

In this study, we show for the first time more dramatic structural differences between the centrioles within one organism, which entail a complete loss of centriole MT. In many experiments attempts have been made to disassemble MT of centrioles, but MT of centrioles are extremely stable and resistant to any treatments. Therefore, experimental attempts to get centrioles without MT in a living cell have never led to success. Even in isolated centrioles, MT were disassembled only when exposed to high concentrations of salt, but the cylindrical shape of the centriole was preserved even under these conditions (Fais et al., 1986).

Finally, during the development of *A. calandrae*, we found three morphological types of centrioles: (i) short MT-free centrioles in larval somatic cells, (ii) short centrioles with MT triplets in the wall of the centriolar cylinder in somatic cells of pupae and adults, (iii) long centrioles with MT triplets in the male germ cells of pupae and adults (Fig. 3; Fig. S3). Unlike in *Drosophila* sperm (Avidor-Reiss et al., 2015), we have never seen the proximal centriole-like structure (PCL) near the basal body of flagella in wasps of *A. calandrae* (this work) and *C. congregata* (Uzbekov et al., 2017). The third type of centriole also differed from the second type by the absence of a cartwheel structure in the lumen of the centriolar cylinder. The cartwheel structure is an obligatory component during early stages of the ninefold central symmetry of centriole formation. We did not find such cartwheel structure in the lumen of type 1 and 3 centrioles. Since this structure was found in procentrioles, we can assume that its absence in ‘adult’ centrioles is associated with its unusually rapid disassembly during the cell cycle in somatic cells of larvae and male germ cells of adult insects. It is also important to note that the absence of the cartwheel structure was observed in both centrioles in the cell, one of which was at least one cell cycle older than the other (Figs 2C and 5H,I,M,N).

In our previous publications (Uzbekov et al., 2017, 2018), we described in detail spermiogenesis in the wasp *C. congregata* and showed that in the process of transformation from spermatids to the mature spermatozoon, the centriole at the base of the flagella is replaced by the cogwheel structure (Fig. S4). The morphology and size of this structure is almost identical to the cogwheel structure described in this paper (Fig. S5). Thus, we can assume that in the process of individual development in wasps there are successive transformations of the cogwheel structure to the centriole and then to the basal body in the spermatids flagellum (Fig. S6).

Exceptions to biological rules often give scientists more information than ‘normal’ organisms. The morphology, biochemistry and development of organisms with mutant genes (that is, departures from normal development) allow novel understanding of the role of these genes in the body. The formation and functioning of centrioles without MT, as described in our paper, is undoubtedly one example of unusual deviation from the typical work of the genetic program. Future studies can examine which genes are activated or repressed to cause this deviation.

## MATERIALS AND METHODS

### Insects

All insects are parasitoid wasps, with larvae developing by the consumption of a living insect host. *A.* *calandrae* (Hymenoptera, Pteromalidae) was reared on its host *Callosobruchus maculatus* (Coleoptera, Bruchidae) in the laboratory under controlled conditions. *N.* *vitripennis* (Hymenoptera, Pteromalidae) was reared on its host *Calliphora* sp. pupae (Diptera, Calliphoridae) in the laboratory under controlled conditions. *C.* *congregata* (Hymenoptera, Braconidae) was reared on its host *Manduca sexta* (Lepidoptera, Sphingidae) in the laboratory under controlled conditions (Beckage et al., 1994). As in all hymenoptera, males are haploid and females are diploid. Experimental males were obtained from unmated egg-laying females.

### Transmission electron microscopy

Larvae and pupae samples were collected at the surface of their host (*Anisopteromalus* and *Nasonia*) or extracted from the bodies of the host (*Cotesia*) before fixation. Imago males were dissected in a drop of phosphate buffer solution (PBS, pH 7.4) after decapitation. All samples were fixed by incubation for 48 h in a mixture of 2% paraformaldehyde and 2% glutaraldehyde in 0.1 M cacodylate buffer (pH 7.4) with 0.1 M sucrose. Samples were then post-fixed by incubation for 1 h with 2% osmium tetroxide in 0.1 M cacodylate buffer (Electron Microscopy Science, Hatfield, USA) with 0.1 M sucrose. Samples were then washed in 0.1 M cacodylate buffer (10 min) and water (3×10 min), dehydrated in a graded series of ethanol solutions (50% for 2×10 min, 70% for 3×15 min, 90% for 3×20 min, 100% for 3×20 min) and propylene oxide (100% for 3×20 min), and embedded in Epon resin (Sigma-Aldrich), which was allowed to polymerize (24 h at 37°C, 48 h at 60°C).

Semi-thin sections (500 nm thick) were cut with a Leica Ultracut UCT ultramicrotome, stained with Toluidine Blue for 30 s at 60°C, washed with distilled water for 5 s, 100% ethanol for 10 s, and again with distilled water for 20 s. They were dried at 60°C and embedded in Epon resin that was allowed to polymerize for 48 h at 60°C. These sections were used for the correct preparation of the analysis zone during the ultrastructural study.

The serial ultra-thin sections (70 nm thick) were cut with a Leica Ultracut UCT ultramicrotome, stained with 5% Uranyl Acetate (20 min) and placed on electron microscopy one-slot grids coated with Formvar film. The sections were observed at 100 kV with a JEM 1011 transmission electron microscope (JEOL, Tokyo, Japan) connected to a Gatan digital camera driven by Digital Micrograph software (Gatan, Pleasanton, USA) for image acquisition and analysis.

### Three-dimensional reconstruction

We recently described the use of serial EM sections for three-dimensional reconstruction (Depla et al., 2010; Ferraris et al., 2013). We used a similar approach here, to generate three-dimensional reconstruction centrioles. Photoshop CS3 software was used to align images from consecutive serial ultrathin section stacks (70 nm thick). Contours were drawn with IMOD software and then arranged into objects. The contours of each object were then joined using the IMOD mesh feature, to form a three-dimensional model.

### Statistical analysis

Plotting and calculation of the standard deviation and *t*-test analysis to statistically assess the reliability of differences in size between different components of centrioles were made using Microsoft Office Excel 2007 software.

## Supplementary Material

Supplementary information
