# Supplementary material for: Centrioles without microtubules: a new morphological type of centriole
Source: Biol Open. 2018 Jul 11;7(8):bio036012. doi: 10.1242/bio.036012 (PMC6124565; doi:10.1242/bio.036012)
Supplement: Supplementary information [file biolopen-7-036012-s1.pdf]

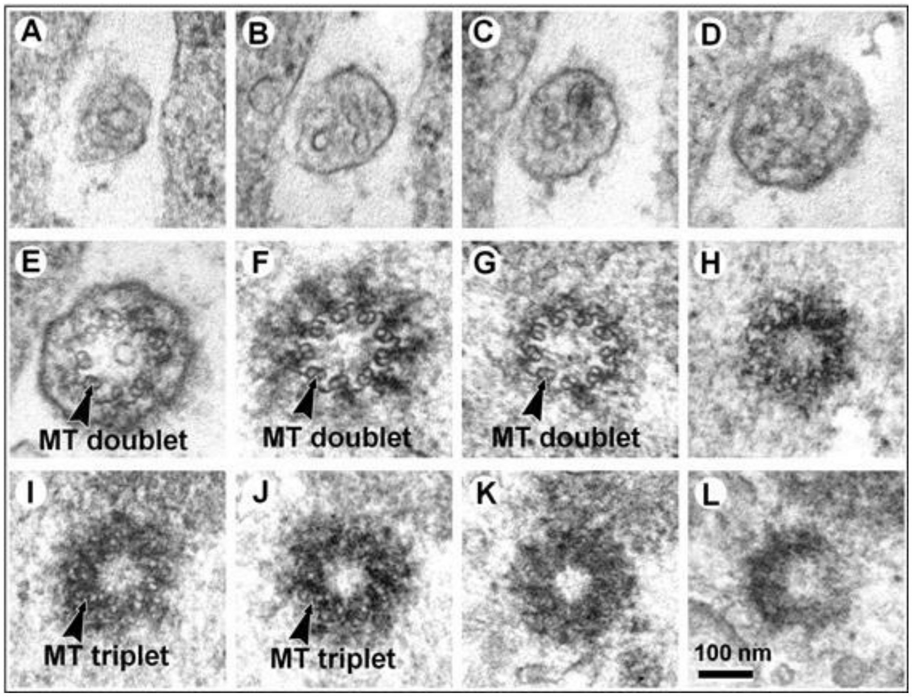

Figure S1. Centriole and primary cilia structure in adult *Anisopteromalus calandrae* generative cell (spermatid). View from the top of primary cilia (view from the distal end of centriole): **(A-D)** top of primary cilia with an irregular structure; **(E-G)** sections of primary cilia with a regular structure; **(H)** section of intermediate zone; **(I-L)** serial sections across the centriole. Scale bar: 100 nm.

Biology Open • Supplementary information

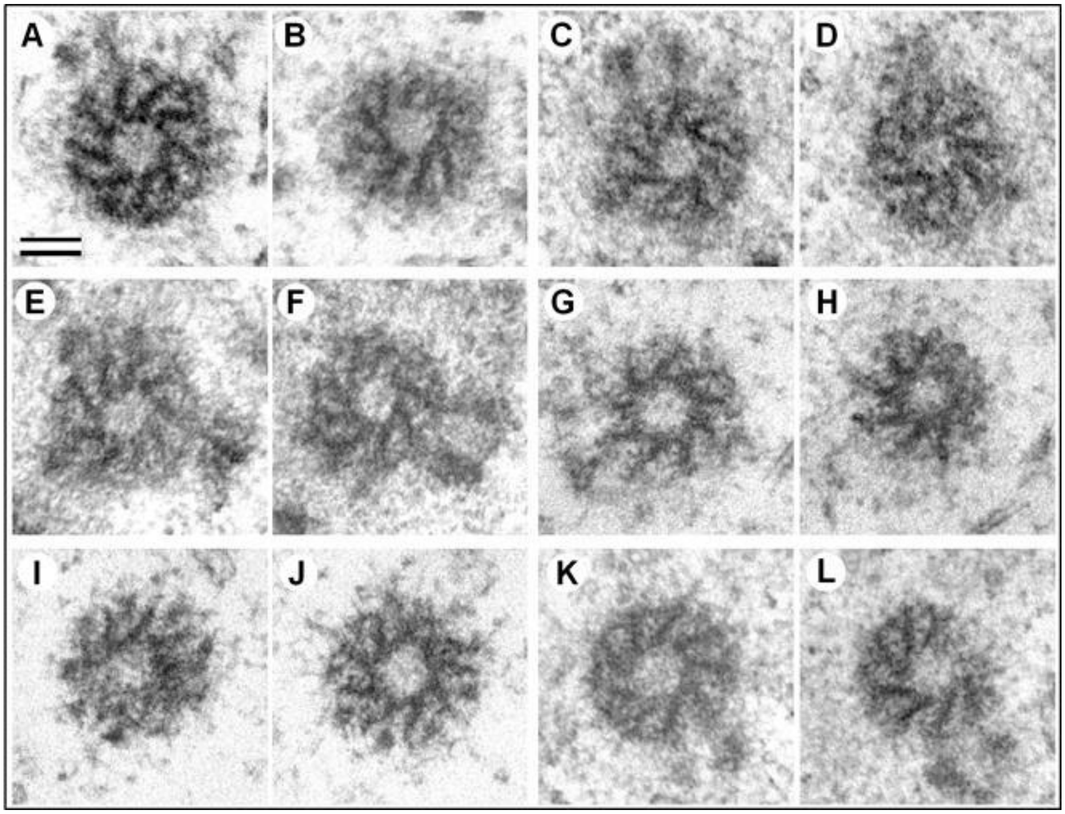

Figure S2. Centriole structure in larvae *Anisopteromalus calandrae* somatic cells. Six different centrioles on cross sections; view from the distal ends of centrioles. (**A, B**) two consecutive sections from centriole 1; (**C, D**) two consecutive sections from centriole 2; (**E, F**) two consecutive sections from centriole 3; (**G, H**) two consecutive sections from centriole 4; (**I, J**) two consecutive sections from centriole 5; (**K, L**) two consecutive sections from centriole 6. Scale bar: 100 nm.

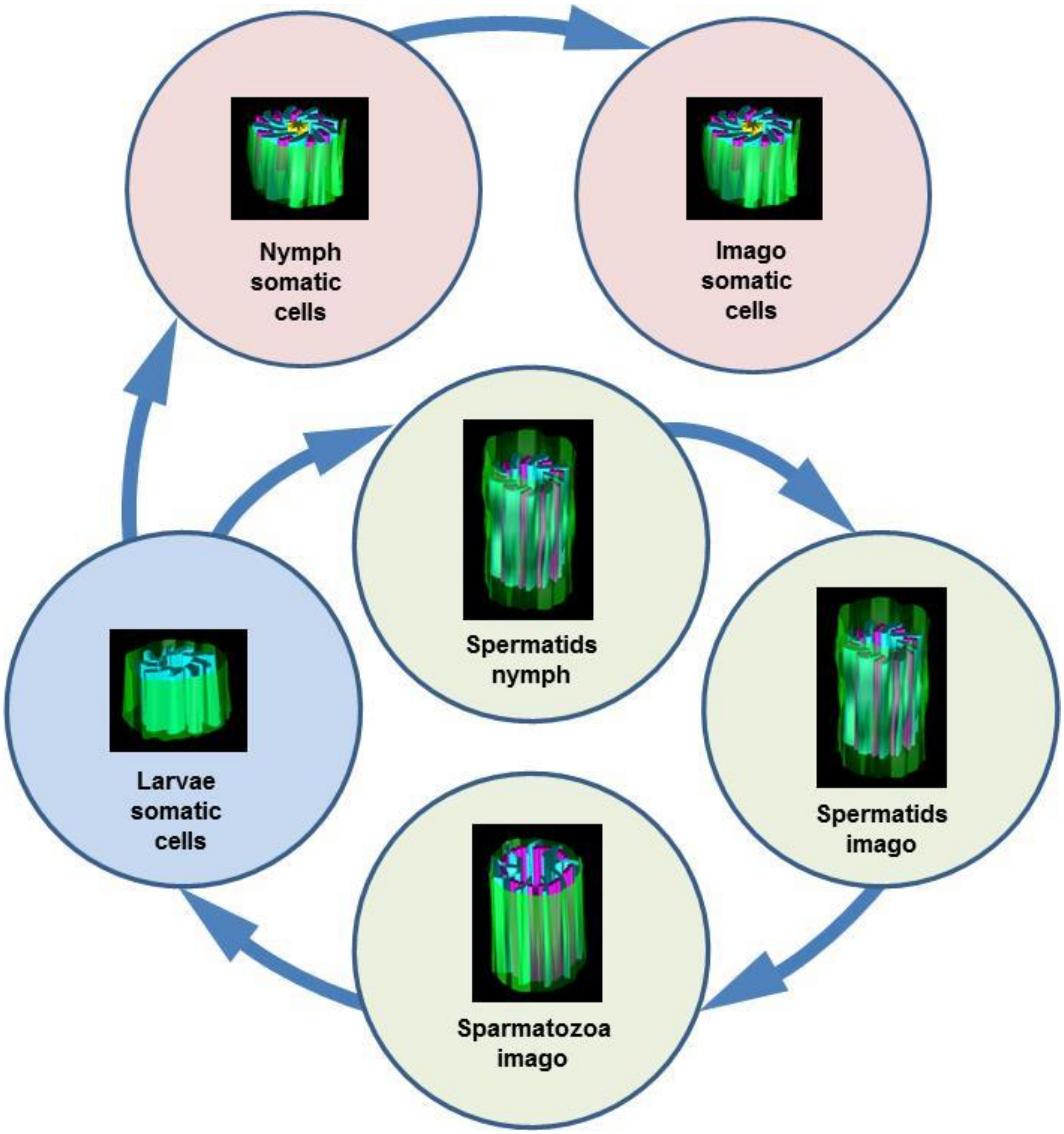

Figure S3. Scheme of mutual transitions of different types of centrioles during *Anisopteromalus calandrae* development. Three morphological types of centrioles: 1) short MT-free centrioles in larval somatic cells (blue circle); 2) short centrioles with MT triplets in the wall of the centriolar cylinder in somatic cells of pupae and adults (pink-brown circles); 3) long centrioles with MT triplets in the male germ cells of pupae and adults (green circles).

Biology Open • Supplementary information

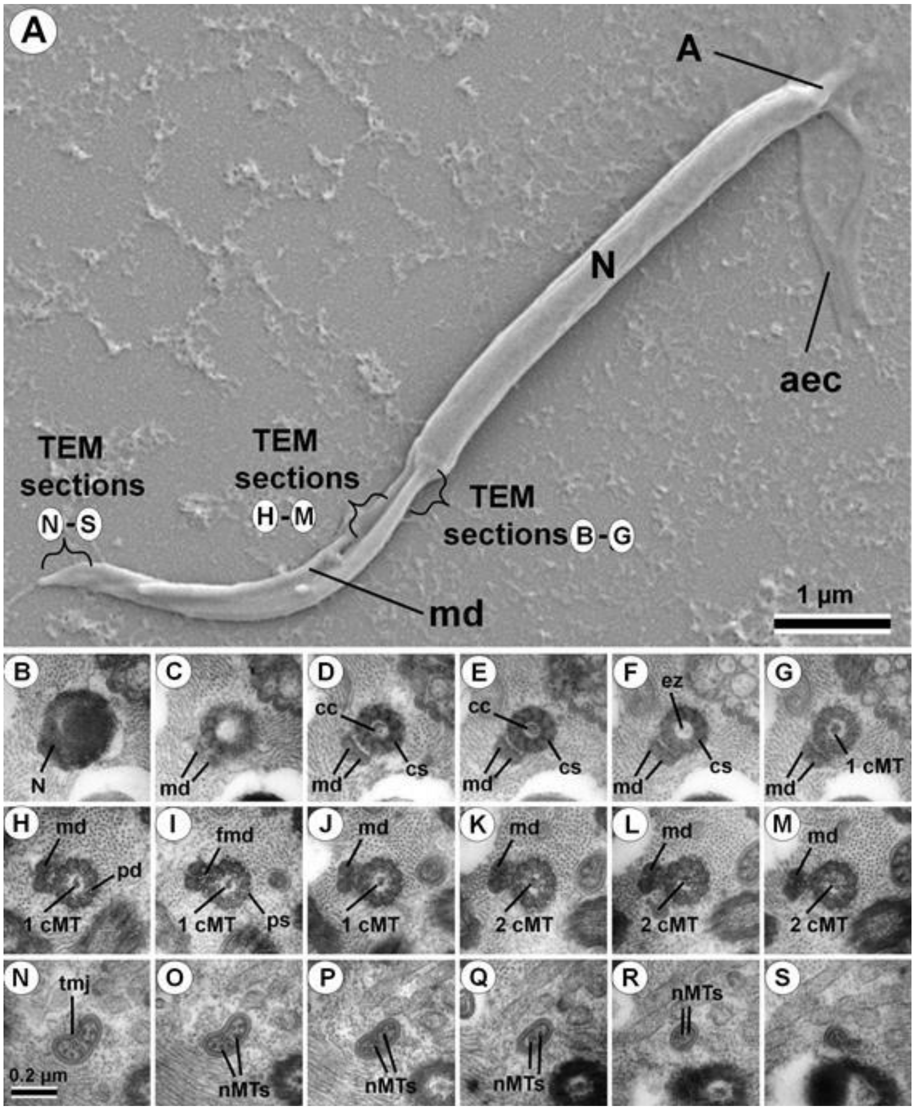

Figure S4. General structure of *Cotesia congregata* mature spermatozoa flagellum.

(**A**) – scanning electron microscopy photo of the sperm on the surface of glass, level of ultrathin cross sections (**B-S**) observed using transmission electron microscopy are shown; (**B-G**) - region from the nucleus to the beginning of first central MT appearance with "cogwheel structure"; (**H-M**) – region from the beginning of first central MT appearance to the beginning of second central MT appearance; (**N-S**) – terminal part of flagellum. A – acrosome; aec - acrosomal extracellular coat; cc – central column of "cogwheel structure"; cs – "cogwheel structure" with 9 prongs; ez – "empty zone" between central column of "cogwheel structure" and the beginning of first central MT; fmd – foot of mitochondrial derivates; md – mitochondrial derivates; N – nucleus; nMTs - non-organized MT of the terminal part of the flagellum; pd – peripheral doublet of MTs; ps – peripheral singlet of MTs; tmj – terminal membrane jacket; 1 cMT – one central MT; 2 cMT – two central MT. Transmission electron microscopy. View from the proximal end of centriole. Scale bar: a - 1 μm, b-s - 0.2 μm. From Uzbekov et al., 2018, with small modifications.

Biology Open • Supplementary information

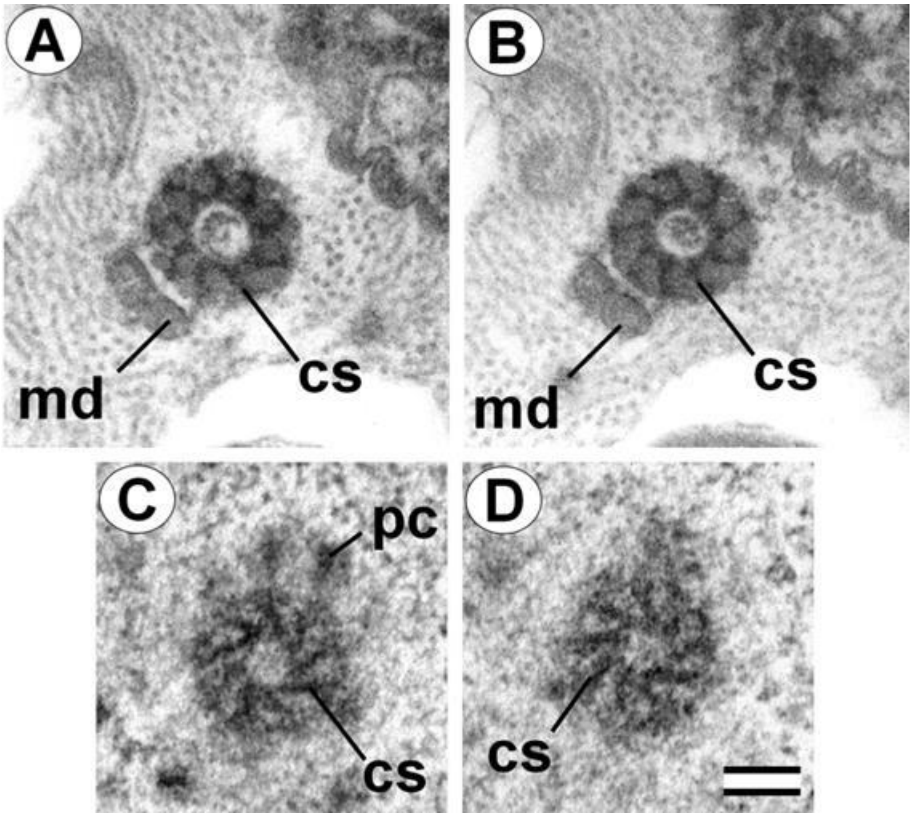

Figure S5. Cogwheel structure in *Cotesia congregata* spermatozoa (A, B) and in *Anisopteromalus calandrae* larvae somatic cells (C, D). cs – "cogwheel structure" with 9 prongs; md – mitochondrial derivates; pc – procentriole. Transmission electron microscopy. View from the proximal end of centriole. Scale bar 100 nm. From Uzbekov et al., 2018, with small modifications.

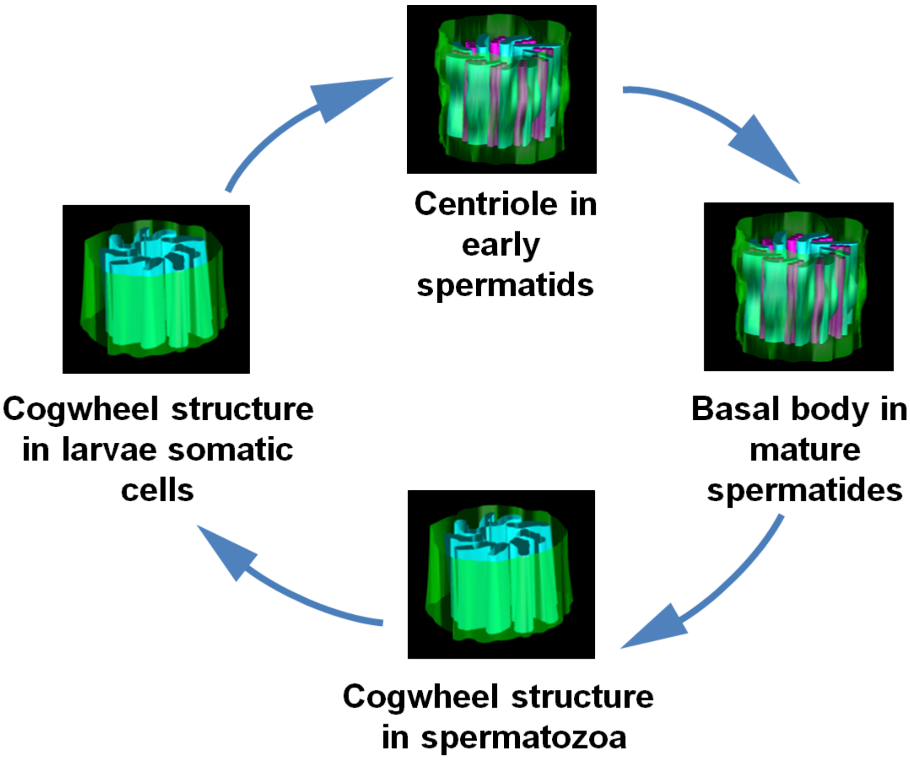

Figure S6. Hypothetical scheme of centriole – basal body - cogwheel structure mutual transformations during wasps living cycle. From Uzbekov et al., 2018.

Biology Open • Supplementary information
